# The association of serum total bile acid with non-alcoholic fatty liver disease in Chinese adults: a cross sectional study

**DOI:** 10.1186/s12944-020-1201-6

**Published:** 2020-02-04

**Authors:** Ziyu Zhang, Wen Dai, Shuwei Weng, Mengdie Luo, Jiahao Fu, John A. Zadroga, Stefano Spolitu, Daoquan Peng

**Affiliations:** 1 Department of Cardiology, The Second Xiangya Hospital, Central South University, 139 Middle Renmin Road, Changsha, 410011 China; 2 National Key Laboratory of Human Factors Engineering, 1 west Yuanmin Yuan Road, Beijing, 100094 China; 3 Rowan University School of Osteopathic Medicine, 1 Medical Center Drive, Stratford, NJ 08084-1501 USA; 4 Department of Medicine, Columbia University, 630 west 168th street, New York, 10032 USA

**Keywords:** Non-alcoholic fatty liver disease, Bile acid, Farnesoid X receptor, Takeda G-protein-coupled receptor 5, Type 2 diabetes, Ziyu Zhang and Wen Dai contributed equally to this work and were listed co-first authors.

## Abstract

**Background:**

Non-alcoholic fatty liver disease (NAFLD) is currently the major cause of chronic liver disease globally. Bile acids (BAs) have emerged as relevant signaling molecules that are associated with NAFLD development. This study was aimed to examine the association of serum total bile acids (TBAs) with NAFLD in a large population of Chinese subjects.

**Methods:**

This cross sectional study recruited 152,336 participants from the Second Xiangya Hospital, China. NAFLD was diagnosed based on the presence of hepatic steatosis on ultrasonography, without significant alcohol consumption and other known causes for chronic liver disease. A multivariate logistic regression model was used to test for the association of serum TBAs with NAFLD, adjusting for conventional risk factors of NAFLD.

**Results:**

A total of 27.4% of the participants had NAFLD. Patients with NAFLD had slightly higher TBA levels than those without, 3.4 vs. 3.0 μmol/L (*p* < 0.001). However, TBA levels were not associated with NAFLD in the multivariate logistic regression model, which adjusted for age, gender and other acknowledged risk factors for NAFLD (OR = 1.00. 95% CI: 1.00–1.00, *p* = 0.797).

**Conclusions:**

We found that the serum TBA levels were not associated with NAFLD. Future studies in a large population, focusing on serum BA composition may improve the understating of the role of BAs in NAFLD.

## Background

Non-alcoholic fatty liver disease (NAFLD) is the hepatic manifestation of metabolic syndrome and is currently the major cause of chronic liver disease globally [1–3]. The estimated prevalence of NAFLD worldwide is approximately 25% in the general population [4]. NAFLD includes a broad clinical and histological spectrum ranging from simple hepatic steatosis (HS) to non-alcoholic steatohepatitis (NASH), with varying degrees of inflammation and fibrosis, which can progress to cirrhosis. It has been reported that approximately 20–30% of subjects with HS would develop NASH [5]. Moreover, NASH has become the leading cause of liver failure and the major indication for liver transplantation [6–9].

Bile acids (BAs) are amphipathic steroid acids that are derived from hepatic cholesterol catabolism in a series of enzyme-catalyzed reactions [10]. Upon synthesis, BAs are subsequently secreted and drained into the gallbladder for temporary storage via the biliary ducts. Postprandial contraction of the gallbladder releases BAs to the duodenum. In the intestine, BAs facilitate the emulsification and absorption of dietary lipids, as well as lipophilic vitamins [10]. After reaching the terminal ileum, BAs are almost completely (95%) reabsorbed and recirculated to the liver via the portal vein. The reabsorbed BAs are secreted back into the bile ducts together with newly synthesized BAs. This process is known as bile acid enterohepatic circulation, which allows for efficient recovery and reuse of the vast majority of BAs [10]. However, a small fraction of reabsorbed BAs bypass enterohepatic circulation and reach the systemic circulation [10].

Interestingly, circulating BAs have emerged as relevant signaling molecules, which act on hepatic and extrahepatic tissues to regulate lipid and carbohydrate metabolic pathways, as well as energy homeostasis, via activation or modulation of bile acid receptors, such as the farnesoid X receptor (FXR) and Takeda G-protein-coupled receptor 5 (TGR5) [11–14]. It has been reported that the BA-FXR/TGR5 pathways regulate hepatic lipid and glucose metabolism, as well as inflammatory activity [15–19].

Moreover, recent studies have shown that blood BAs are closely associated with NAFLD in humans. Serum total bile acid (TBA) levels in NAFLD patients (*n* = 16) were approximately 3 times higher than those of healthy controls (*n* = 11) [20]. Similarly, Rebecca et al. found that patients with type 2 diabetes (T2D), a major risk factor for NAFLD, had a nearly two-fold elevation in their plasma TBA [21].

A major limitation of these studies was their small sample sizes and to our knowledge, the association between serum TBAs and NAFLD has not been examined in a large-scale epidemiologic study before. Thus, this study was aimed to evaluate whether serum TBA levels were independently associated with NAFLD in a large population of Chinese subjects.

## Methods

### Study design and population

In this cross-sectional study, participants were recruited from the Health Management Center, The Second Xiangya Hospital, Changsha, China, from 2010 to 2017. We included adult participants who were scheduled to receive systemic medical evaluation, including subjective medical history, physical exam, blood chemistries, electrocardiogram and abdominal ultrasound. Subjects were excluded if they 1) were less than 18 years old; 2) reported excess alcohol consumption or a history of viral hepatitis, autoimmune hepatitis, or other known causes of chronic liver disease. 152,336 subjects were ultimately included in this study. This study was approved by the ethics committee institutional review board of The Second Xiangya Hospital of Central South University. The study was carried out in accordance with the 1975 Declaration of Helsinki and pertinent regulations. Informed written consent was obtained from all participants.

### Data collection

Demographic characteristics, medical histories, blood chemistries and abdominal ultrasound data were gathered for all participants at the time of enrollment. Specific blood chemistries included fasting serum BAs, glucose and lipids, as well as liver and renal function tests. As previously described [22, 23], blood samples were drawn by venipuncture after overnight fasting. The blood specimens were processed and measured at the central laboratory in The Second Xiangya hospital, which is standardized and certified. An automatic biochemistry analyzer (Hitachi 7360; Hitachi Ltd., Tokyo, Japan) and commercially available kits were used to measure serum biochemistries according to accompanying manuals. Specifically, serum BAs were measured using enzymatic assay [24].

NAFLD was diagnosed based on the evidence of fatty liver upon abdominal ultrasonography and the exclusion of other known etiology of chronic liver diseases [25]. Hypertension was referred to as blood pressure ≥ 140/90 mmHg in more than two measurements and/or the requirement of or treatment with anti-hypertension drugs [26]. Diabetes was diagnosed as the existence of fasting serum glucose levels ≥7.0 mmol/L, and/or random serum glucose ≥11.1 mmol/L, and/or 2-h post-prandial serum glucose ≥11.1 mmol/L on oral glucose tolerance test (OGTT) in multiple measurements and/or the requirement of treatment with hypoglycemic agents [27]. Patients were diagnosed with coronary artery disease (CAD) based on the clinical manifestation, such as chest pain, and evidences indicating myocardial ischemia [28, 29].

### Statistical analysis

Numerical variables were expressed as the mean (standard deviation) or as medians (Q1-Q3 quartiles), depending on the pattern of data distribution. Categorical variables were expressed as percentages (numbers). Differences in numerical variables between groups were analyzed by the independent *t* test, analysis of the variance (ANOVA), Mann-Whitney *U* test or Kruskal-Wallis *H* test, as appropriate. Differences in categorical variables were analyzed by the chi-square test. Logistic regression analyses were performed to examine the association of serum TBA with NAFLD and T2D. SPSS software (version 20.0; SPSS Inc., Cary, Chicago, USA) was used to perform the statistical analyses. For all analyses, two-tailed *p* values < 0.05 were considered statistically significant.

## Results

### Clinical characteristics

The demographic and clinical characteristics of the study population were shown in Table 1. The mean age and body mass index (BMI) of participants was 45 years old and 24.0 kg/m^2^, respectively, and 54.2% (82,533/152,336) were males. 27.4% (41,771/152,336) of individuals were diagnosed with NAFLD. We also found 19.5% (29,767/152,336), 3.7% (5623/152,336) and 1.3% (1920/152,336) of participants had hypertension, T2D and CAD, respectively. Individuals with NAFLD were noted to be older, have a higher BMI and increased prevalence of hypertension, T2D and CAD than those without (all *p* < 0.001).

**Table 1:** Demographic and clinic characteristics of the study population

	Total (*n* = 152,336)	With NAFLD (*n* = 41,771)	Without NAFLD (*n* = 110,565)	p
Clinical characteristics				
Age, years	45 (13)	48 (12)	44 (14)	<.001
Male percentage, % (n)	54.2% (82,533)	75.4% (31,510)	46.1% (51,023)	<.001
BMI, kg/m^2^	24.0 (3.3)	27.1 (2.8)	22.9 (2.8)	<.001
Systolic pressure, mm Hg	120 (17)	128 (16)	117 (17)	<.001
Diastolic pressure, mm Hg	76 (11)	81 (11)	74 (10)	<.001
Hypertension, % (n)	19.5% (29,767)	33.8% (14,129)	14.1% (15,638)	<.001
T2D, % (n)	3.7% (5623)	6.9% (2870)	2.5% (2753)	<.001
CAD, % (n)	1.3% (1920)	1.8% (733)	1.1% (1187)	<.001
Biochemical parameters				
TBA, μmol/L	2.0 (3.1–5.0)	3.4 (2.3–5.4)	3.0 (1.9–4.8)	<.001
AST, U/L	23 (17)	26 (14)	21 (17)	<.001
ALT, U/L	26 (26)	37 (28)	22 (24)	<.001
BUN, mmol/L	5.0 (1.4)	5.2 (1.4)	5.0 (1.5)	<.001
Cr, μmol/L	67.3 (22.6)	71.9 (19.0)	65.6 (23.6)	<.001
UA, μmol/L	312.3 (83.1)	359.3 (81.2)	294.6 (75.8)	<.001
FBG, mmol/L	5.04 (1.29)	5.48 (1.76)	4.90 (1.01)	<.001
OGTT-2 h BG †, mmol/L	7.10 (3.07)	7.74 (3.45)	6.74 (2.77)	<.001
TC, mmol/L	4.66 (0.93)	4.92 (1.00)	4.56 (0.88)	<.001
HDLC, mmol/L	1.31 (0.30)	1.16 (0.24)	1.37 (0.30)	<.001
LDLC, mmol/L	2.67 (0.77)	2.81 (0.83)	2.62 (0.74)	<.001
RLP-C, mmol/L	0.68 (0.61)	0.95 (0.88)	0.57 (0.43)	<.001
TG, mmol/L	1.24 (0.86–1.86)	1.87 (1.33–2.74)	1.07 (0.78–1.53)	<.001

Data are shown as mean (standard deviation), median (Q1–Q3 quartiles), or percentages (n). *P* values from analysis of the independent t test, Mann-Whitney *U* test, or chi-square test. Two-tailed *p* < 0.05 was considered statistically significant. *BMI* body mass index, *NALFD* non-alcoholic fatty liver disease, *T2D* type 2 diabetes, *CAD* coronary artery disease, *TBA* total bile acid, *FBG* fasting blood glucose, *AST* aspartate aminotransferase, *ALT* alanine aminotransferase, *BUN* blood urea nitrogen, *Cr* creatinine, *UA* uric acid, *TC* total cholesterol, *LDL-C* LDL cholesterol, *HDL-C* HDL cholesterol, *RLP-C* remnant lipoprotein cholesterol, *TG* triglyceride. *OGTT-2 h BG* oral glucose tolerance test 2 h-post blood glucose. † 7870 individuals had the OGTT-2 h data

### Serum TBA levels

**Fig. 1:**
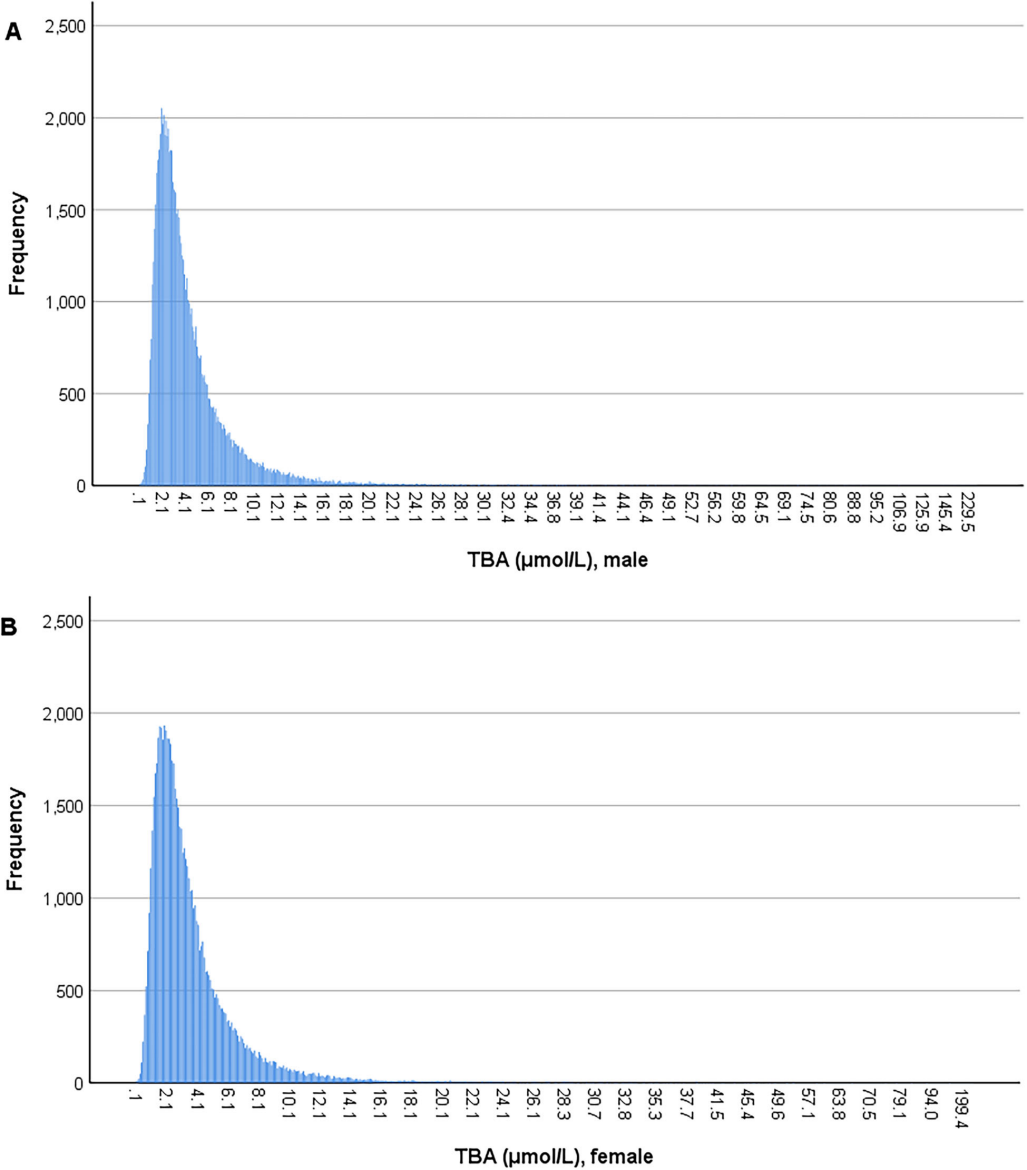
Distribution of serum total bile acid levels in males and females. **a**. distribution of serum total bile acid levels in males, **b**. distribution of serum total bile acid levels in females

Consistent with previous findings [30], the distribution of serum TBA levels was positively skewed in both male and female populations in our study (Figure 1). The median level of TBA was 3.4 μmol/L in males, which was significantly higher than the level of 2.8 μmol/L in females (*p* < 0.001). Moreover, this was applied to all age strata in the study population (Table 2). Currently, the reference range of TBA is less than 10 μmol/L for both males and females in the clinical setting. According to this criterion, 6.2% of males and 3.5% of females had elevated TBA in this study.

**Table 2:** Gender and age stratum specific median levels of serum TBA

Age	Male		Female		p
	n	TBA, μmol/L	n	TBA, μmol/L	
< 20	189	2.9 (1.9–4.6)	179	3.0 (1.8–5.1)	< 0.01
20~29	9099	3.4 (2.2–5.4)	10,283	2.9 (1.9–4.5)	< 0.01
30~39	18,094	3.2 (2.1–5.1)	16,062	2.6 (1.7–4.1)	< 0.01
40~49	22,738	3.3 (2.1–5.2)	18,851	2.7 (1.7–4.2)	< 0.01
50~59	18,852	3.4 (2.2–5.4)	15,243	2.9 (1.9–4.7)	< 0.01
60~69	8377	3.6 (2.4–5.8)	6445	3.2 (2.1–5.2)	< 0.01
≥ 70	5184	4.2 (2.8–6.9)	2740	3.9 (2.5–6.2)	< 0.01
Total	82,533	3.4 (2.2–5.4)	69,803	2.8 (1.8–4.5)	< 0.01

Data are shown as median (Q1–Q3 quartiles). *P* values from analysis of the Kruskal-Wallis H test. Two-tailed *p* < 0.05 was considered statistically significant. *TBA* total bile acid

### Serum TBA and NAFLD

Patients with NAFLD were noted to have slightly higher TBA levels than those without (3.4 vs. 3.0 μmol/L, *p* < 0.001) (Table 1). We then calculated the prevalence of NAFLD categorized by TBA quintiles and we found that the prevalence of NAFLD increased with the elevation in TBA levels. The 5th quintile (Q5) group had the highest (31.3%) while the 1st quintile (Q1) group had the lowest prevalence (20.8%) of NAFLD (Table 3). Furthermore, we used a multivariate logistic regression model to test whether serum TBA was associated with NAFLD after adjusting for age, gender, BMI and other acknowledged risk factors for NAFLD. After this adjustment, we found that the association between serum TBA level and NAFLD was lost (Table 4).

**Table 3:** Basic characteristics of the study population categorized by TBA quintiles

	Serum TBA quintiles					P
	Q1 (*n* = 31,267)	Q2 (*n* = 30,136)	Q3 (*n* = 31,298)	Q4 (*n* = 29,245)	Q5 (*n* = 30,390)	
Clinical characteristics						
Age, years	44 (12)	45 (13)	45 (13)	46 (14)	48 (15)	<.001
Male percentage, % (n)	42.5% (13,284)	52.0% (15,679)	55.7% (17,428)	58.6% (17,129)	62.6% (19,013)	<.001
BMI, kg/m2	23.7 (3.3)	24.0 (3.3)	24.1 (3.4)	24.2 (3.4)	24.3 (3.4)	<.001
Systolic pressure, mm Hg	117 (17)	119 (17)	120 (17)	121 (17)	123 (18)	<.001
Diastolic pressure, mm Hg	74 (11)	75 (11)	76 (11)	76 (11)	77 (11)	<.001
NALFD, % (n)	20.8% (6512)	26.0% (7840)	28.8% (9012)	30.4% (8895)	31.3% (9512)	<.001
Hypertension, % (n)	14.7% (4581)	17.6% (5309)	19.3% (6028)	21.9% (6403)	24.5% (7446)	<.001
T2D, % (n)	2.0% (630)	3.1% (931)	3.7% (1161)	4.6% (1358)	5.1% (1543)	<.001
CAD, % (n)	0.9% (269)	0.9% (282)	1.2% (387)	1.4% (407)	1.9% (575)	<.001
Biochemical parameters						<.001
AST, U/L	20 (7)	21 (9)	22 (9)	23 (10)	26 (32)	<.001
ALT, U/L	22 (14)	25 (18)	26 (20)	27 (22)	32 (44)	<.001
BUN, mmol/L	4.8 (1.4)	4.9 (1.4)	5.1 (1.5)	5.1 (1.5)	5.1 (1.5)	<.001
Cr, μmol/L	64.6 (21.7)	66.7 (21.5)	67.9 (26.6)	68.3 (21.7)	69.2 (20.5)	<.001
UA, μmol/L	299.1 (80.3)	310.4 (82.6)	314.7 (83.2)	317.3 (83.8)	320.6 (84.2)	<.001
FBG, mmol/L	4.90 (1.01)	4.98 (1.18)	5.04 (1.31)	5.11 (1.41)	5.16 (1.49)	<.001
TC, mmol/L	4.65 (0.91)	4.66 (0.92)	4.66 (0.92)	4.67 (0.95)	4.66 (0.97)	0.103
HDLC, mmol/L	1.35 (0.29)	1.32 (0.29)	1.30 (0.29)	1.29 (0.30)	1.28 (0.31)	<.001
LDLC, mmol/L	2.70 (0.76)	2.69 (0.77)	2.68 (0.77)	2.67 (0.78)	2.62 (0.78)	<.001
RLP-C, mmol/L	0.60 (0.47)	0.65 (0.56)	0.68 (0.60)	0.71 (0.66)	0.76 (0.74)	<.001
TG, mmol/L	1.11 (0.80–1.61)	1.21 (0.84–1.81)	1.25 (0.87–1.90)	1.30 (0.89–1.97)	1.34 (0.92–2.06)	<.001

Data are shown as mean (standard deviation), median (Q1–Q3 quartiles), or percentages (n). *P* values from analysis of the ANOVA, Kruskal-Wallis *H* test, or chi-square test. Two-tailed *p* < 0.05 was considered statistically significant. *BMI* body mass index, *NALFD* non-alcoholic fatty liver disease, *T2D* type 2 diabetes, *CAD* coronary artery disease, *TBA* total bile acid, *FBG* fasting blood glucose, *AST* aspartate aminotransferase, *ALT* alanine aminotransferase, *BUN* blood urea nitrogen, *Cr* creatinine, *UA* uric acid, *TC* total cholesterol, *LDL-C* LDL cholesterol, *HDL-C* HDL cholesterol, *RLP-C* remnant lipoprotein cholesterol, *TG* triglyceride

**Table 4:** The association of serum TBA with NAFLD by multivariate logistic regression analysis

Variables	OR	95% CI	p
Age	1.02	1.01–1.02	< 0.001
Gender (male vs. female)	1.47	1.42–1..52	< 0.001
BMI	1.59	1.58–1.60	< 0.001
History of hypertension (with vs. without)	1.28	1.23–1.33	< 0.001
History of T2D (with vs. without)	1.58	1.48–1.70	< 0.001
TC	0.98	0.92–1.04	0.501
LDL-C	1.39	1.31–1.48	< 0.001
HDL-C	0.38	0.34–0.42	< 0.001
TG	1.35	1.31–1.39	< 0.001
TBA	1.00	1.00–1.00	0.797

*P* values were from multivariate logistic regression. Two-tailed *p* < 0.05 was considered statistically significant. *TBA* total bile acid, *NAFLD* non-alcoholic fatty liver disease, *BMI* body mass index, *T2D* type 2 diabetes, *TC* total cholesterol, *LDL-C* LDL cholesterol, *HDL-C* HDL cholesterol, *TG* triglyceride

### Serum TBA and T2D

Since NAFLD is closely associated with T2D and insulin resistance is considered one of the major cause for NAFLD development, we also checked the association of serum TBA with T2D. Similarly, we found that the prevalence of T2D rose with increasing TBA levels. Notably, the Q5 group had the highest and Q1 group had the lowest prevalence of T2D, 5.1% vs. 2.0%, respectively (Table 3). However, in our multivariate logistic regression analysis, serum TBA was not associated with T2D in the model controlling for age, gender, BMI and known T2D risk factors (Table 5).

**Table 5:** The association of serum TBA with T2D by multivariate logistic regression analysis

Variables	OR	95% CI	p
Age	1.07	1.07–1.07	< 0.001
Gender (male vs. female)	1.33	1.25–1.42	< 0.001
BMI	1.01	1.00–1.02	0.136
History of hypertension (with vs. without)	1.61	1.52–1.72	< 0.001
History of NAFLD (with vs. without)	1.73	1.62–1.85	< 0.001
TC	1.04	0.97–1.11	0.253
LDL-C	0.88	0.82–0.95	< 0.001
HDL-C	0.34	0.29–0.39	< 0.001
TG	1.06	1.04–1.09	< 0.001
TBA	1.00	1.00–1.01	0.178

*P* values were from multivariate logistic regression. Two-tailed *p* < 0.05 was considered statistically significant. *TBA* total bile acid, *NAFLD* non-alcoholic fatty liver disease, *BMI* body mass index, *T2D* type 2 diabetes, *TC* total cholesterol, *LDL-C* LDL cholesterol, *HDL-C* HDL cholesterol, *TG* triglyceride

## Discussion

The understandings of the function and effect of BA modulated receptors in the liver, such as FXR, suggest the potential role of BAs in NAFLD development. However, in this study, we found that serum TBA levels were not independently associated with NAFLD or T2D when controlling for age, gender, BMI and additional risk factors.

BAs are a group of diverse amphipathic steroids, rather than a single type of molecule, derived from the catabolism of cholesterol in hepatocytes in a process that involves two principal pathways [10]. The classical pathway, which generates the majority of BAs, is regulated by the rate-limiting enzyme, cholesterol 7α-hydroxylase (CYP7A1). The alternative pathway is initially catalyzed by sterol-27-hydroxlase (CYP27A1), followed by BA hydroxylation through oxysterol 7α-hydroxylase (CYP7B1). These two pathways account for the synthesis of the primary BAs, namely chenodeoxycholic acid (CDCA) and cholic acid (CA). They, in turn, are modified by gut microbiota to yield lithocholic acid (LCA) and deoxycholic acid (DCA) respectively, which represent two major type of secondary BAs.

Different types of BAs differ in in their physicochemical properties. For example, whereas CDCA is a potent agonist of FXR, DCA and LCA have been suggested as partial antagonists of FXR [10]. This suggests they may have a distinct regulatory effect on metabolism. Recent findings indicated that alterations in circulating BA composition were closely associated with NAFLD and T2D. Na et al. measured the serum BA composition of NAFLD patients and healthy controls and found that the percentage of FXR antagonistic DCA was increased, while the agonistic CDCA was decreased in patients with NAFLD compared to healthy subjects [20]. Rebecca et al. showed that increased plasma levels of 12a-hydroxylated BA were associated with insulin resistance in both T2D patients and healthy subjects [21]. These interesting findings suggest that the alterations in BA composition rather than quantitative changes in TBA levels are more relevant to metabolic homeostasis.

Currently, no convenient/applicable serum BA composition assessment techniques are available in non-academic clinics. So far, penetration of "classical" laboratory methods, including high-performance liquid chromatography (HPLC), liquid chromatography-mass spectrometry (LC-MS) and gas chromatography-mass spectrometry (GC-MS), into the clinics is minimal due to the need of expensive equipment and time-consuming protocols [24]. Because of that, we had to resort to the conventional enzymatic assessment of blood TBA in this study, which is widely used in clinical settings

In addition to this, we acknowledge several other limitations to our study. Due to the nature of cross-sectional studies, we were not able to follow participants to determine the predictive values of serum TBA levels in NAFLD development. Besides, we only measured the fasting serum BA levels. The role of postprandial BA levels in metabolism should also be studied in the future.

## Conclusion

Serum TBA was not associated with NAFLD. Future studies in a large population focusing on the serum BA composition may strengthen our understating of the role of BAs in NAFLD development and progression.

## Data Availability

The datasets used and/or analyzed during the current study are available from the corresponding author on reasonable request.

## Abbreviations

ANOVA: Analysis of the variance
BAs: Bile acids
BMI: Body mass index
CA: Cholic acid
CAD: Coronary artery disease
CDCA: Chenodeoxycholic acid
CYP27A1: Sterol-27-hydroxlase
CYP7A1: Cholesterol 7α-hydroxylase
CYP7B1: Oxysterol 7α-hydroxylase
DCA: Deoxycholic acid
FXR: Farnesoid X receptor
GC-MS: Gas chromatography-mass spectrometry
HPLC: High-performance liquid chromatography
HS: Hepatic steatosis
LCA: Lithocholic acid
LC-MS: Liquid chromatography-mass spectrometry
NAFLD: Non-alcoholic fatty liver disease
NASH: Non-alcoholic steatohepatitis
OGTT: Oral glucose tolerance test
T2D: Type 2 diabetes
TBA: Total bile acid
TGR5: Takeda G-protein-coupled receptor 5
